# Endothelial cyst of the adrenal gland: A surgical case report

**DOI:** 10.1016/j.ijscr.2024.109334

**Published:** 2024-02-02

**Authors:** Ahmed Ibrahimi, Salim Ouskri, Jihad Lakssir, Hachem El Sayegh, Yassine Nouini

**Affiliations:** IBN SINA Hospital, Morocco

**Keywords:** Adrenal, Cyst, Endothelial

## Abstract

**Introduction and importance:**

Advances in medical imaging have led to increased detection of rare adrenal cysts, typically asymptomatic. The article emphasizes their silent nature and the evolving diagnostic methods.

**Case presentation:**

Woman underwent successful laparoscopic removal of a 9 cm left adrenal cyst. Histological analysis identified it as an endothelial cyst.

**Clinical discussion:**

Adrenal cysts, mostly unilateral, have diverse histological types. Diagnosis relies on imaging, and surgical intervention is required for symptomatic cases or those exceeding 5 cm. Minimally invasive techniques have improved outcomes.

**Conclusions:**

Adrenal cysts, generally benign and asymptomatic, pose diagnostic challenges. Surgical intervention is recommended for larger or symptomatic lesions, with advancements in minimally invasive options improving outcomes.

## Highlights

• Adrenal cysts, rare but increasingly detected. Often asymptomatic, posing a diagnostic challenge
• 41-year-old female, history of hypertension
• Abdominal ultrasound and CT reveal a large adrenal cyst.
• Adrenal cysts primarily unilateral, female prevalence
• Diagnosis relies on imaging and histological examination and management depends on symptoms; surgical intervention.

## 1 Introduction

Adrenal cysts, although rare, are increasingly detected due to advances in modern medical imaging, leading to the increased identification of cystic incidentalomas [1]. These cysts are typically considered incidental findings as they are most often silent, causing few to no clinical manifestations in patients [2,3]. In this article, we detail the rarity of this pathology and the evolution of diagnostic means for detecting these adrenal lesions [1].

Their rarity is largely due to the fact that these cysts tend to remain asymptomatic, meaning that patients may live with them for many years without even being aware [4,5]. However, advances in imaging techniques have enabled more precise identification of these lesions, revealing a significant diagnostic and management challenge [4].

## 2 Case presentation

It's the case of a 41 years-old female patient, with a history of high blood pressure for the last 3 years revealed by vertigo. The patient was initially treated with captopril and subsequently with atenolol. Following a year of antihypertensive therapy, treatment resistance led to the worsening of her high blood pressure, prompting further investigations to determine the cause.

Clinical examination revealed slight overweight (BMI: 25,7 kg/m2), high blood pressure at 20/10 mmHg, and tenderness in the left lumbar region.

**Fig. 1:**
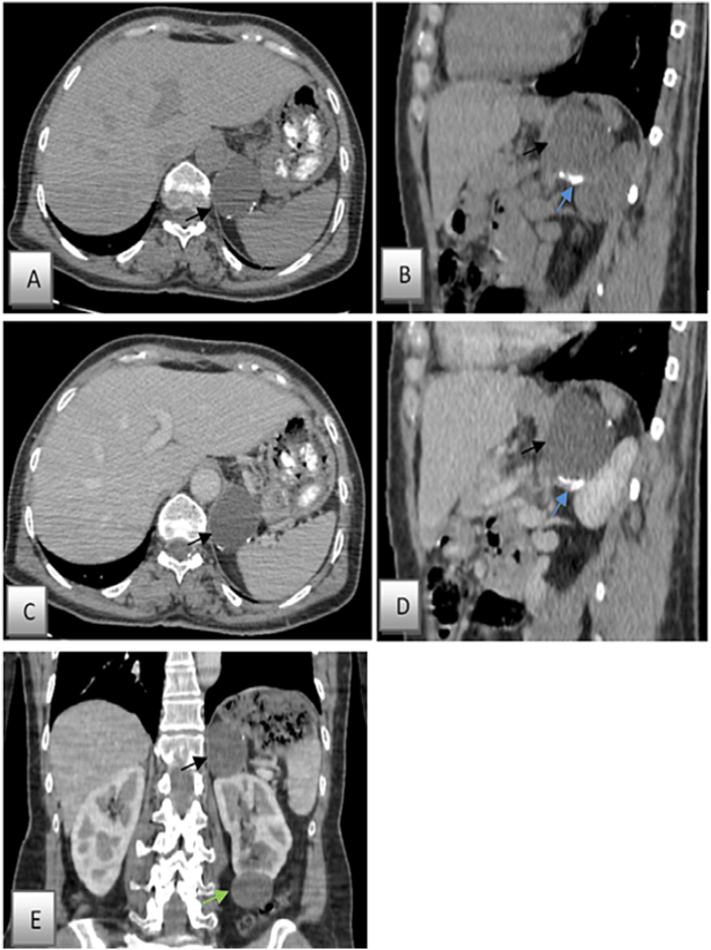
Non contrast-enhanced abdominal CT scan images (Fig. A: axial, Fig. B: sagittal), and non enhanced images (Fig. C: axial, Fig. D: sagittal, Fig.E: coronal), revealing a large well-limited cystic, homogeneous mass of the left adrenal gland (black arrow), with regular contours, containing some linear parietal calcifications (blue arrow), not septated, not enhanced after injection of contrast agent, without any rupture or intracystic hemorrhage. The mass measured 4.5 × 4.4 × 5 cm in cranio-caudal, antero-posterior and transverse dimensions and was abutting various parts of the gastrointestinal tract and the kidney.

An abdominal ultrasound was performed and did not reveal any kidney anomalies but indicated the presence of a heterogeneous process located near in the upper pole of the left kidney, suggesting an adrenal lesion. Computed tomography of the adrenal glands confirmed the presence of a heterogeneous round-shaped left adrenal mass, measuring 5 cm in diameter, with well-defined and regular margins. No signs of local or regional invasion were detected, and no retro-peritoneal adenomegaly was reported. A clear limit with the left kidney was evident. The lesion exhibited an uneven texture with small calcifications and a central area with a liquid density (Fig. 1).

The contralateral adrenal gland was of normal size and appearance. The blood and urine adrenal hormonal work-up, did not reveal anomalies indicative of a secretory tumor.

**Fig. 2:**
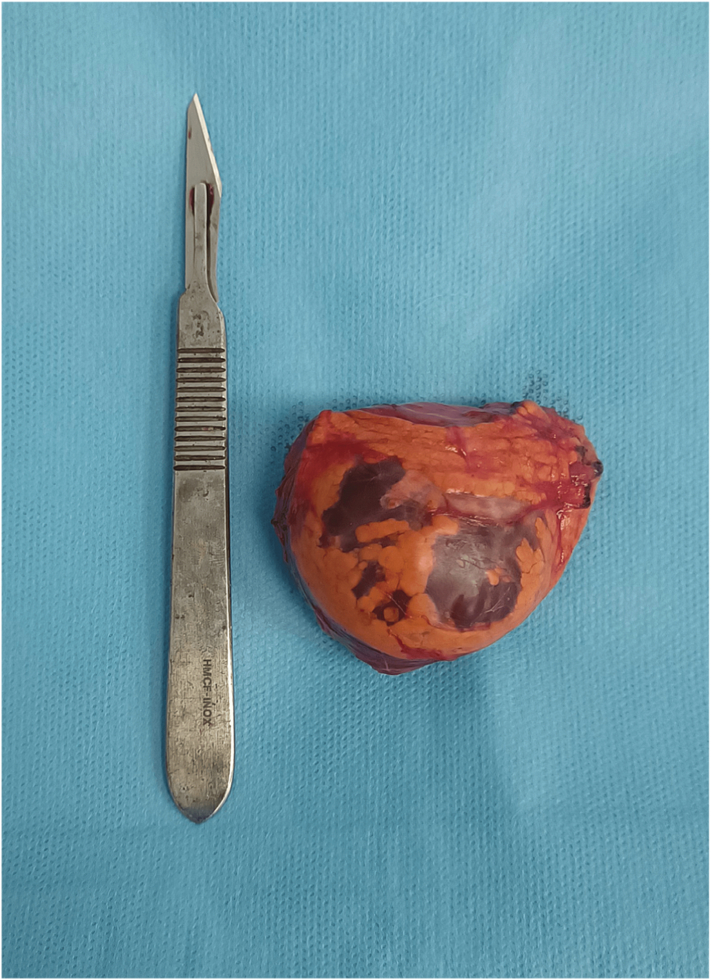
Operative piece image showing the cystic lesion within the adrenal parenchyma.

In light of the imaging data, a transperitoneal laparoscopic adrenalectomy was chosen as the appropriate treatment modality using three trocars, with a placement identical to a laparoscopic nephrectomy but slightly displaced upward for better exposure. The dissection of the adrenal gland was straightforward, and the entire adrenal gland with the cyst was removed without aspiration of the cystic content allowing the removal of a 60 g gland, predominantly consisting of a well-contained cyst with chylous fluid content (Fig. 2).

The performance of a cystic enucleation was not feasible as the cyst occupied the entire gland without preservable parenchyma.

The postoperative recovery was uneventful, and the patient was discharged on the first post-operative day.

**Fig. 3:**
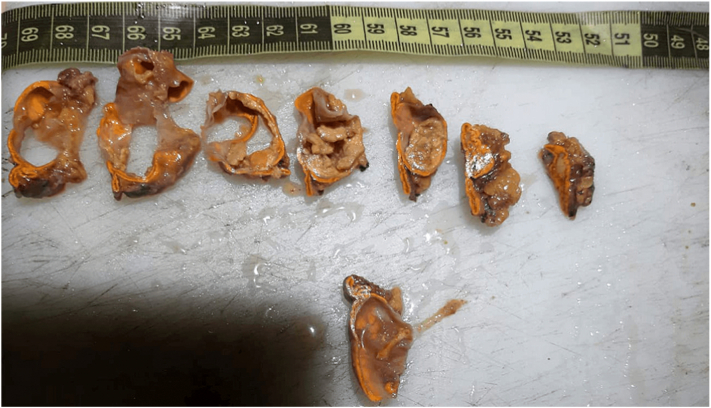
Macroscopic view of the cyst.

**Fig. 4:**
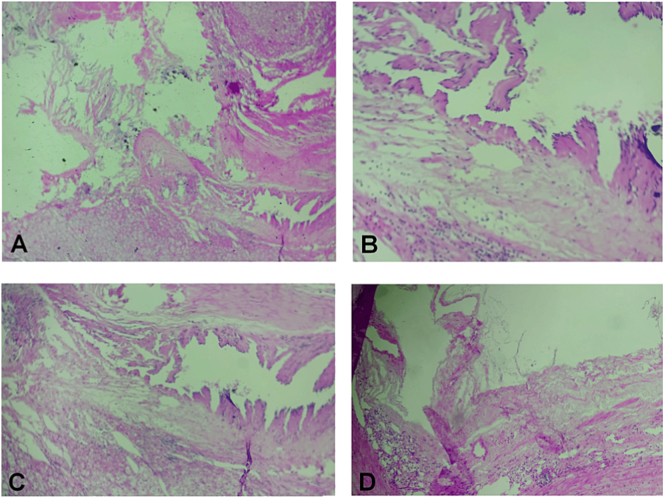
Adrenal cyst's wall lined by endothelial like cells. A + D: Low power. B: High power. C: Medium power showing the epithelial cells.

Histological analysis identified the cyst as an endothelial cyst with no signs of malignancy (Fig. 3, Fig. 4).

The postoperative recovery proceeded without complications, and the patient was discharged on the first postoperative day, with a three-month follow-up showing moderate hypertension (16/9 cm Hg) effectively controlled with an ACE inhibitor witch was the main indication for the operation.

## 3 Discussion

Adrenal cysts are usually observed unilaterally, although bilateral forms have been reported in up to 8 % of autopsies. Their size varies considerably, reaching up to 12 cm in diameter. Histologically, four main types of cysts are distinguished: parasitic, epithelial, pseudocysts, and endothelial, each having distinct histological characteristics [1,3,[5], [6], [7], [8]].

- Parasitic cysts: They represent 7% of cases and are related to hydatid disease, with a confirmed diagnosis by the presence of parasites or their constituents inside the cyst [9].
- Epithelial cysts: Account for 9 % of cases and have an epithelial layer. Their origin remains debated, although links to embryonic processes are suggested [1,8].
- Pseudocysts: Found in 39 % of cases, these cysts are characterized by a fibrous wall without epithelial or endothelial structure. Their origin is varied, possibly resulting from hemorrhagic, ischemic, infectious processes, or tumor degeneration [1,7,8].
- Endothelial cysts: Constitute 45 % of cases and are lined with endothelium, containing chylous or hemorrhagic content. Calcifications are observed in 15 % of cases, and they are more often lymphangiomas than angiomas. These lesions can be hamartomas or indicative of lymphatic or vascular stasis [1,8].

These cysts are present at any age, but their peak frequency is between 50 and 70 years, with a female predominance [8]. Though often asymptomatic, large or complex adrenal cysts can cause abdominal pain, sepsis, or hemodynamic failure [6]. The association with high blood pressure (HBP), once rare, seems to be increasing likely due to diagnostic advancements [1]. However, the relationship between cysts and HBP remains debated, although cases of blood pressure normalization after cyst resection have been reported [1,6].

The diagnosis of adrenal cysts relies on a combination of imaging and biological information, although confirmation often occurs during histological examination after the operation [4]. Measurements of adrenal hormones and their derivatives do not conclusively rule out the presence of a secreting tumor, such as pheochromocytomas [1,8].

Pseudocysts observed in the adrenal gland may indicate potential tumor transformation, which questions the benign nature of these cysts. Therefore, a cautious approach is necessary for their management.

Symptomatic cases, whether related to clinical symptoms, excessive hormonal secretion, or infections, require surgical intervention. This can involve simple cyst removal (cystectomy) or more commonly, complete removal of the adrenal gland (adrenalectomy) [3,5].

For asymptomatic cystic lesions, management is similar to other incidental adrenal findings. It largely depends on their size. The decision to intervene is often based on a threshold diameter, usually ranging between 3 and 6 cm [3,5]. There is a debate regarding the usefulness of percutaneous puncture before potential surgical intervention. A watchful approach involves strict monitoring, primarily based on imaging (computed tomography). Lesion progression is a key argument for a more aggressive management [3,6].

The choice of surgical approach largely depends on the pathological context and, to a lesser extent, the patient's morphology [3,5,6]. Lesions considered benign are generally accessible by lombotomy, a vertical incision in the back, or intercostal. Conversely, suspicions of pheochromocytoma warrant larger incisions, notably thoraco-phrenolaparotomy, to avoid abrupt manipulation of the adrenal gland. Lesions suggestive of necrotic corticosurrenaloma are often treated through laparotomy, especially a subcostal incision, to facilitate tumor exposure and removal [3,5].

## 4 Conclusions

Adrenal cysts are a group of rare lesions that are generally benign and asymptomatic. However, diagnosing and managing them can be difficult due to their heterogeneous appearance and the challenge of determining the pathological subtype during routine imaging tests. It is essential to rule out malignancy and understand the functional status in managing these lesions. Any functional, potentially malignant lesion should undergo surgical intervention, while other cases can be managed conservatively. Thanks to advancements in surgical techniques and minimally invasive options, the morbidity and mortality associated with these lesions have been significantly reduced, even when detecting potential malignancy through imaging remains challenging.

## Methods

This work has been reported in line with the SCARE criteria.

## Ethical approval

Ethical approval for this study was provided by the Ethical Committee of IBN SINA University Hospitals, Rabat, Morocco on 10/10/2023.

## Funding

NA.

## Guarantor

Ahmed Ibrahimi
Salim Ouskri.

## CRediT authorship contribution statement

Ahmed Ibrahimi Urology assistant Professor: First author has contributed in the writing and correction the case report
Salim Ouskri Urology Resident: second author has contributed in the writing and correction the case report
Jihad Lakssir Urology Resident: has contributed in the correction the case report
Hachem El Sayegh Urology Professor: has contributed in the correction the case report
Yassine Nouini Urology Professor: has contributed in the correction the case report.

## Declaration of competing interest

NA.
